# Further assessment of the non-cognitive adaptive resourcefulness model comprising mental toughness, resilience, and self-efficacy: relationships with emotional intelligence and chronic time pressure

**DOI:** 10.3389/fpsyg.2026.1718213

**Published:** 2026-02-24

**Authors:** Andrew Denovan, Danny Powell, Neil Dagnall

**Affiliations:** 1 School of Psychology, Liverpool John Moores University, Liverpool, United Kingdom; 2 School of Psychology, Manchester Metropolitan University, Manchester, United Kingdom

**Keywords:** chronic time pressure, emotional intelligence, mental toughness, non-cognitive skills, optimal regulation, self-efficacy

## Abstract

**Introduction:**

Preceding research by synthesizing overlapping elements of mental toughness, optimal regulation, and self-efficacy identified Non-Cognitive Adaptive Resourcefulness (NCAR): a general, positive psychological energy that enables coping. Although validated by subsequent research, further investigation was needed to nomologically validate NCAR alongside allied non-cognitive skills and assess criterion validity against practical outcomes. To address this, the current study evaluated NCAR in relation to emotional intelligence (EI) and chronic time pressure (CTP).

**Methods:**

This study included 1,007 UK-based respondents (*M*age = 37.30 years, *SD* = 11.65, range 18–66), with 572 females, 429 males, five non-binary, and one who preferred not to disclose gender. Data were collected through an online survey that incorporated multiple psychometric instruments.

**Results:**

Exploratory structural equation modeling (ESEM) successfully replicated the NCAR model and determined that EI was distinct. Specifically, EI shared regulatory components with NCAR but was distinguishable via its focus on emotion identification and understanding. Structural equation modeling revealed that NCAR significantly predicted Feeling Harried (FH), the subjective, stressful experience of being rushed, but showed mixed results for Cognitive Awareness (CA), the objective appraisal of time shortage.

**Discussion:**

Overall, results demonstrated that NCAR emphasizes emotional and coping-related aspects of time perception (FH) while facilitating realistic appraisal of time constraints (CA). Future research should examine the extent to which these findings generalize to other populations, including through cross-cultural comparisons.

## Introduction

Non-cognitive skills encompass a range of personal qualities (i.e., mindsets, attitudes, behaviors, and strategies) not directly related to intellectual abilities. Theorists consider non-cognitive skills beneficial as research documents they facilitate positive psychological functioning (e.g., goal achievement, management of emotions, and interpersonal interactions) (Ren et al., 2019). Concurrently, non-cognitive skills are affiliated with psychological wellbeing. Possession of mental toughness, which enhances stress resistance and reduces depressive symptoms illustrates this (e.g., Papageorgiou et al., 2019; Mojtahedi et al., 2021). Likewise, self-efficacy (Zhang et al., 2024) and ego-resiliency (Kubo et al., 2021) link to good mental health. Furthermore, non-cognitive skills are positively related to enhanced performance across a range of everyday domains, including educational attainment (Clough et al., 2016; Malanchini et al., 2024), sport (Dagnall et al., 2021; Taylor et al., 2025; Wheatley et al., 2023), leadership (Yu et al., 2022), prosocial behavior (e.g., helping, sharing, and cooperating) (Ritgens et al., 2024), and effective life choices (Moffitt et al., 2011).

While there is general academic consensus that possession of non-cognitive skills is personally and socially advantageous, a tendency among researchers to focus exclusively on specific constructs (e.g., mental toughness, resistance, grit, and self-efficacy), has produced a lack of scholarly coherence within the literature. Specifically, studies typically examine constructs in isolation and fail to compare their relative merits or consider the extent to which non-cognitive skills share adaptive qualities. Noting this, Denovan et al. (2023a) adopted a harmonizing, integrated approach. This involved assessing construct overlap between mental toughness, grit, ego resiliency, and self-efficacy to determine the extent to which these non-cognitive skills shared characteristics that promote positive psychological outcomes. Explicitly, coalescence of these non-cognitive skills produced a general adaptive non-cognitive resource that facilitates the ability to withstand stress, pressure and adversity, enables coping, and encourages optimal performance.

Denovan et al. (2023a) selected mental toughness, grit, ego resiliency, and self-efficacy because these constructs feature extensively within non-cognitive literature. Although various definitions of mental toughness exist, the construct denotes a “psychological resource that is purposeful, flexible, and efficient in nature for the enactment and maintenance of goal-directed pursuits” (Gucciardi, 2017, p. 18). Grit is perseverance and passion for long-term goals (Drinkwater et al., 2019), particularly the capacity to actively sustain effort and interest, when confronted by failures and barriers (Liang, 2021). Ego resiliency designates an affect-processing system that allows individuals to flexibly modify their responses to changing situational demands, especially during emotionally challenging conditions (Block, 2002; Wang et al., 2019).

The construct comprises Optimal Regulation (OR) and Openness to Life Experience (OLE). OR is the capacity to appropriately adjust level of self-control to situational demands. This includes regulating impulses, feelings, and desires. OLE reflects inclination to engage with novel and unusual situations. This involves adopting a positive outlook and the capability to adapt to changing environments (Block and Kremen, 1996; Letzring et al., 2005; Smith et al., 2021). Finally, self-efficacy denotes personal belief in competency and facility to achieve desired goals (Bandura, 1997). This belief is an important stress managing resource (Livintti et al., 2021), which empowers individuals to confidently face and overcome challenges.

At the construct level, when confronted with adversity (i.e., challenges, hardships, and difficulties), mental toughness, grit, ego resiliency, and self-efficacy evoke distinct psychological processes. Mental toughness functions as an overarching, initiative-taking tendency that facilitates endurance and growth during times of pressure (Drinkwater et al., 2019; Mojtahedi et al., 2023). This manifests as a positive mindset embodying confidence and commitment, which enables individuals to manage failure and success (Clough et al., 2002; Dagnall et al., 2021; Liew et al., 2019). Ego resiliency is a dynamic and reactive capacity, which denotes the ability to adapt (i.e., emotionally and behaviorally) to the shifting demands of adverse situations. This involves the capacity to recover quickly (i.e., bounce back) from hardship through adjustment (Block and Kremen, 1996; Kubo et al., 2021; Letzring et al., 2005). Grit concerns sustained passion and perseverance to achieve long-term goals, even when faced with setbacks. These attributes reflect endurance and dedication over extended periods (Credé et al., 2017; Duckworth et al., 2007, 2019). Finally, in terms of responding to adversity, self-efficacy provides a targeted resource. Particularly, belief in one’s personal capability to execute actions required to overcome challenges (Bandura, 1997; Caliendo et al., 2023).

In addition to response variations to adversity, scholars have identified dispositional differences between mental toughness, grit, ego resiliency, and self-efficacy. Theorists regard grit and ego resiliency as traits, reflecting stable individual differences in psychological resources (Block and Kremen, 1996; Duckworth et al., 2007), whereas scholars conceptualize mental toughness and self-efficacy as both a stable trait (general belief in competence) and a context-specific state (confidence in ability to successfully complete a particular task) (Bandura, 1997; Liew et al., 2019). In the case of mental toughness, theorists explain this variation using the terms plastic or malleable trait, whereby though largely fixed it is possible to increase construct level through development (Clough et al., 2021; Gucciardi, 2017). Evidence to support this notion comes from behavioral genetics, where studies report that half of the variance in mental toughness is attributable to non-shared environmental factors (e.g., Horsburgh et al., 2009; Veselka et al., 2009). Of the 4Cs factors, Commitment and Control possess the lowest heritability suggesting that these dimensions are trainable.

While conceptually important, distinguishing between constructs offers limited practical utility, as individuals simultaneously employ various non-cognitive skills. In this context, examining commonality between distinct but potentially related non-cognitive constructs has theoretical and applied benefits.

Noting this, Denovan et al. (2023a) assessed the mutual properties of mental toughness, grit, ego resiliency, and self-efficacy. In study one, exploratory structural equation modeling (ESEM) bifactor analysis found that mental toughness, self-efficacy, and optimal regulation subscale of ego-resilience loaded onto a single, general factor. Thus, the emergent construct reflected the capacity to adapt to challenges and utilize internal resources, Denovan et al. (2023a) named this non-cognitive adaptive resourcefulness (NCAR).

Explicitly NCAR embodied a positive mindset (i.e., collection of beliefs, attitudes, and assumptions that structure how an individual sees the world, themselves, and others), which enables coping. Coping in the context of NCAR denotes managing both the demands of failure and success (Denovan et al., 2023a). The broadening of coping to incorporate positive factors alongside negative circumstances distinguishes NCAR from non-cognitive skills which focus on adversity (e.g., resilience and hardiness). For instance, students manage anxieties/concerns arising from deadlines and lower than desired marks and the exigencies of maintaining exacting standards of academic performance. Thus, NCAR better reflects the dynamic nature of real-world contexts, which require flexibility and the ability to manage a range of situations/circumstances. The dynamic nature of complex environments necessitates a mindset capable of addressing diverse, fluctuating challenges concurrently, thereby offering a comprehensive approach to coping.

Illustrating the distinct features of NCAR, Grit (perseverance and passion for long-term goals; Drinkwater et al., 2019) and Openness to Life Experiences (skilled expressiveness and diligence; Klohnen, 1996) failed to load on to the model. This reinforced the notion that NCAR focuses on dynamic psychological management rather than sustained effort or broad expressiveness.

The active features of NCAR embody the properties of its constituent elements. Mental toughness contributes a flexible psychological resource that facilitates effective goal-directed performance (Gucciardi, 2017). Perceived self-efficacy emphasizes the psychological importance of self-belief (Waddington, 2023). Additionally, optimal regulation reflects the crucial need to manage psychological functioning when facing stressors (Dębski et al., 2021).

The robust nature of the NCAR model emerged through study two replication, which revealed satisfactory invariance. This indicated that respondents interpreted items similarly across both studies. Denovan et al. (2023b) extended NCAR validation (see Denovan et al., 2023a) by appraising alternative solutions (i.e., multidimensional and one-factor solutions) and testing nomological validity (via NCAR relationships with perceived stress and anxiety control). Demonstrating that NCAR correlates in theoretically predictable ways with related constructs (perceived stress and anxiety control) evidences nomological validity. Since composite constructs ally to effective handling of pressure and higher emotional management (i.e., mental toughness, Gucciardi, 2017; adaptive self-regulation from ego-resilience; Kołodziej-Zaleska et al., 2023; self-efficacy; Zhang et al., 2024) high NCAR should prognosticate reduced perceived stress and increased anxiety control. Thus, testing such relationships helps to confirm NCAR’s validity.

ESEM found that a bifactor (vs. one-factor and multi-factor alternatives) solution represented data more effectively than one-factor and multi-factor alternatives. This model comprised an overarching general factor and subfactors corresponding to the composite constructs (mental toughness, optimal regulation, and self-efficacy). As the researchers anticipated, NCAR significantly predicted perceived stress and anxiety. Indeed, NCAR predicted perceived stress to a greater extent than the specific bifactors. This suggested that NCAR captured an underpinning, positive psychological energy that facilitates coping. Particularly, an enabling resource that enhances the capacity to thrive under pressure and retain emotional control in demanding and trying circumstances. Additionally, bifactors retained unique predictive capacity. Illustratively, mental toughness demonstrated a stronger predictive relationship than NCAR. This occurred because optimal regulation and self-efficacy contain aspects that weaken the predictive capacity of NCAR relative to stress and anxiety control. This was because mental toughness indexes stress/anxiety-specific features, whereas optimal regulation (i.e., ability to balance psychological functioning) and self-efficacy (i.e., self-belief) assess general, non-specific propensities (see Dębski et al., 2021; Farkas and Orosz, 2015; Schwarzer and Jerusalem, 1995).

### The present study

This study extended previous research by establishing the generalizability of NCAR and broadening assessment of nomological validity. Replicating the construct model in an independent sample was crucial to confirm the factorial structure and ensure construct consistency beyond preceding studies. To achieve this, the authors assessed latent structure using a bifactor model. Following this, to further evaluate nomological validity, analysis evaluated the additive role of emotional intelligence (EI; the ability to perceive, understand, and manage emotions). Analysis determined whether EI meaningfully contributed to the model and whether its dimensions overlapped with, or diverged from, the specific facets of NCAR.

The authors selected EI as the construct’s theoretical underpinnings suggest a strong relationship with adaptive functioning, particularly in managing emotions and navigating social challenges (Haag et al., 2025). These features are integral to NCAR’s conceptualization of resilience and adaptation. From a broad conceptual perspective, assessing NCAR relative to other non-cognitive skills including EI represents a logical progression of determining precisely how NCAR is located within the general sphere of non-cognitive skills. For instance, whether it represents a distinct cluster of related skills / constructs, or a general dimension that extends across other non-cognitive skills. Previous research thus far supports the former (e.g., Denovan et al., 2023a). Due to possessing overlapping qualities, EI features including emotion regulation and management should align more closely with NCAR. For instance, shared features of emotion regulation with MT and OR, and management (e.g., of responses to stress/demands) with self-efficacy.

To assess criterion validity, the authors regressed the NCAR model onto Chronic Time Pressure (CTP). CTP refers to a persistent and pervasive feeling of being rushed and having insufficient time to complete tasks or meet demands. It represents a state where individuals consistently experience a lack of time, leading to anxiety and potential negative impacts on well-being. Development of CTP was informed by the work of researchers such as Szollos (2009) and Roxburgh (2002, 2004). Theoretically, NCAR should relate (negatively) to CTP by virtue of embodying a positive psychological mindset that enables active coping (Denovan et al., 2023b).

Conducting this analysis determines criterion validity by assessing whether the model can account for or estimate characteristics of behavior exhibited in specific situations (i.e., under time pressure), thus extending the previous analyses of Denovan et al. (2023b). Consistent with prior research, the investigators specified CTP as a bifactor ESEM model, featuring a general factor alongside Cognitive Awareness of Time Shortage (CA) and Feeling Harried (FH). This allowed the researchers to determine the NCAR’s criterion utility against a theoretically relevant real-world stressor. Particularly, how NCAR components relate to and predict experiences of time pressure.

## Materials and methods

### Sample

The sample consisted of 1,007 UK-based participants, *M*age = 37.30 years, *SD* = 11.65, range 18–66. Specifically, 572 females, *M*age = 34.82 years, *SD* = 10.54, range 18–64; 429 males, *M*age = 40.67 years, *SD* = 12.22, range 20–66; five non-binary, *M* = 29.0 years, *SD* = 6.67, range 19–37; and one who preferred not to disclose gender (53 years of age). With regards to ethnicity, 840 identified as White, 39 as Black, 86 Asian, 25 Mixed ethnic background, nine preferred to not disclose, and eight identified as Other ethnicity. Recruitment was via Bilendi Ltd. The exclusion criterion was that individuals must be at least 18 years of age. The researchers requested a representative sample from Bilendi. This used UK benchmarks from the 2021 Census data for England and Wales (Office for National Statistics, 2021). Specifically, in addition to a reasonable sample size (>1,000), a slightly greater quota of females than males (56% vs. 43%), a mean age close to 40, and over 81% identifying as White, 9% Asian, 4% Black, and 2% Other ethnicity. Data collected via panels provides high quality data equivalent to traditional recruitment approaches (Kees et al., 2017). Bilendi collates data from a pre-arranged pool of individuals consenting to take part in survey-based research. In this sense, the sample is opportunity-based.

### Measures

#### Mental toughness

The 48-item Mental Toughness Questionnaire (MTQ-48) assesses the capacity to handle pressure and recover from setbacks via four primary factors: Control, Commitment, Challenge, and Confidence (Clough et al., 2002). Due to the assessment of multiple constructs, the present study used the abridged, unidimensional, 10-item version (MTQ-10). The researchers selected the MTQ-10 in preference to the original abridged version, the MTQ-18 (Clough et al., 2002) because it is psychometrically superior (Dagnall et al., 2019). Items within MTQ measures appear as statements (e.g., “I generally feel in control”) and participants indicate their responses on a five-point Likert type scale (1 = Strongly Disagree to 5 = Strongly Agree). Higher scores indicate greater levels of mental toughness. Research has demonstrated robust psychometric properties for this scale (e.g., Dagnall et al., 2019; Denovan et al., 2021, 2024a; Papageorgiou et al., 2018).

#### Optimal regulation

The Optimal Regulation (OR) subscale from the Ego Resiliency Scale (ER89) (Block and Kremen, 1996) assessed qualities related to insight, confidence, and warmth, including the capacity to maintain homeostasis of the personality system in response to stressors/difficulties (Dębski et al., 2021). OR is a core feature of ego resiliency (Alessandri et al., 2012), alongside Openness to Life Experiences (i.e., productive activity and skilled expressiveness) (see Alessandri et al., 2007). The scale presents items as statements (e.g., “I quickly get over and recover from being startled”) and participants indicate their level of endorsement via a four-point Likert type scale (1 = Does Not Apply at All to 4 = Applies Very Strongly). Good psychometric properties exist for the OR subscale (Denovan et al., 2022a, 2022b).

#### Self-efficacy

The 10-item General Self-Efficacy Scale (GSES) (Schwarzer and Jerusalem, 1995) measured participants’ belief in their ability to cope with life challenges and obtain desired outcomes. Within the GSES, items (e.g., “I am confident that I could deal efficiently with unexpected events”) appear as statements alongside a four-point Likert type response format (1 = not at all true to 4 = exactly true). The GSES is an established instrument, which possesses established psychometric integrity (Zeng et al., 2020).

#### Emotional intelligence

The Brief Emotional Intelligence Scale-10 (BEIS-10) (Davies et al., 2010) is an abridged version of the 33-item emotional intelligence scale developed by Schutte et al. (1998). The measure is a concise self-report questionnaire designed to assess emotional intelligence (EI) in adults. Participants respond to statements related to their emotional intelligence (e.g., “I know why my emotions change”) using a 5-point Likert scale (1 = strongly disagree to 5 = strongly agree). The scale is based on the ability model of EI, developed by Salovey and Mayer (1990), which emphasizes the ability to perceive, understand, and manage emotions. The BEIS-10 has demonstrated good reliability and validity (Durosini et al., 2020; Vredeveld, 2018).

#### Chronic time pressure

The 13-item Chronic Time Pressure Inventory (CTPI) (Denovan and Dagnall, 2019) focuses on the degree to which individuals feel they do not have enough time to do what they need or want to do, and the associated feelings of pressure. Participants respond to items (e.g., “I am often in a hurry”) with a five-point Likert response scale (1 = strongly disagree to 5 = strongly agree). The CTPI includes two related but distinct components: cognitive awareness of time shortage (CA) and feeling harried (FH) (Denovan and Dagnall, 2019). CA captures cognitive awareness of time constraints and FH evaluates affective notions of feeling rushed. Research provides psychometric support for this scale (e.g., Denovan et al., 2023c, 2024b).

### Procedure and ethics

Prospective participants received a web-link accompanied by an information sheet, which described the study background, procedure, ethics details, and sought consent for taking part. The online survey instructed consenting participants to take their time and respond to all questions openly and honestly. The survey further informed participants that questions measured preferences, and that no correct or incorrect responses existed. These instructions worked to limit socially desirable responding.

Study materials comprised an opening demographics section (i.e., age, preferred gender), measures, and a concluding section. The concluding section provided the debrief, reminding participants of the study’s purpose and their rights. To reduce potential order effects, researchers rotated scale presentation across participants. The Manchester Metropolitan University Faculty of Health, Psychology and Social Care Ethics Committee granted ethical authorization (approval #52314).

### Analysis

Analysis progressed through three stages. The first replicated validation study outcomes. This was necessary to establish that the existence of a general non-cognitive factor comprising Mental Toughness, Optimal Regulation, and Self-Efficacy was not an artifact of previous validation studies, and to demonstrate that the model generalized across samples. Correspondingly, analysis evaluated latent structure with a bifactor model. Following guidance from previous research (e.g., Denovan et al., 2024a), specification of MTQ10 included correlating uniqueness among negatively keyed items (i.e., 2, 3, 6, and 7).

The second stage of analysis evaluated the additive role of EI to the Non-Cognitive Adaptive Resourcefulness (NCAR) model by specifying EI as an additional correlated specific factor alongside Mental Toughness, Optimal Regulation, and Self-Efficacy. EI was modeled as oblique to the other specific factors and orthogonal to the general NCAR factor for interpretive clarity. If the EI specific factor accounted for substantial variance in EI items beyond the general factor, this indicated that EI captured unique variance not explained by general non-cognitive resourcefulness, supporting its distinction as a separate construct. Conversely, if EI items loaded weakly on the EI factor but strongly on the general factor, this inferred that EI is subsumed within the general NCAR construct, supporting its integration rather than separation (and hence expansion of NCAR).

Bifactor modeling enabled scrutiny of the degree of multidimensionality, while indicating the configuration and orientation of items loading on the general NCAR factor (Denovan et al., 2023b). In this context, a strength of bifactor modeling is the identification of systematic item variance relative to a general component and sources of supplementary variance, such as bifactors (Rodriguez et al., 2016a, 2016b).

Stage one and two analysis utilized exploratory structural equation modeling (ESEM) via Mplus v8 (Muthén and Muthén, 2018). This assessed item effects across factors by not restricting non-target loadings to zero and permitting cross-loadings (Marsh et al., 2010). Implementation of target loading (oblique rotation) involved allocating zero loadings to items that did not belong to the scale in relation to the model structure, while permitting other items to be free (Schonfeld et al., 2019). The third stage comprised extending previous nomological validity analyses of the NCAR factorial structure. This involved regressing the model onto the criterion measure of Chronic Time Pressure. Following previous analyses (e.g., Denovan et al., 2024b), the researchers specified this as a bifactor ESEM model with a general factor alongside bifactor factors of CA and FH.

Throughout analyses, assessment of data-model fit used chi-square, Comparative Fit Index (CFI), Tucker–Lewis Index (TLI), Standardized Root-Mean-Square Residual (SRMR), and Root-Mean-Square Error of Approximation (RMSEA). Satisfactory fit includes CFI ≥ 0.90, TLI ≥ 0.90, SRMR ≤ 0.08 and RMSEA ≤ 0.08 (Hu and Bentler, 1995). Model computation used the Weighted Least Squares Mean and Variance-adjusted (WLSMV) estimator (theta parameterization; threshold criterion = 0.500D-04). Developers designed WLSMV to handle ordinal data, such as Likert-scale items, by treating the ordinal responses as a result of a categorization process. It provides more accurate estimates of model parameters compared to maximum likelihood, especially when the data is non-normal, and is a recommended method for Likert-scale item analysis (Park, 2023). However, typical model comparison methods for non-nested solutions (e.g., Akaike Information Criterion, AIC) are not possible because WLSMV does not rely on maximum log-likelihood estimation, which is a key component of the AIC calculation. Instead, model comparison focused on surveying fit indices.

Bifactor model interpretation also employed the indices of Rodriguez et al. (2016a, 2016b). At the model level, this considered explained common variance (ECV), hierarchical omega (*ω_h_*), and percentage of uncontaminated correlations (PUC). Reise et al. (2013) concluded that PUC < 0.80, ECV > 0.60 and *ω_h_* > 0.70 is suggestive of a robust general component, and a unidimensional model can be utilized in subsequent structural equation analyses without introducing more than 10% parameter bias. Higher factor determinacy (FD > 0.90) and construct replicability (*H* > 0.80), alongside stronger relative omega (*ω*) for the general vs. bifactors, specify that a construct should be evaluated at a total score as opposed to bifactor level. Nonetheless, since this study was exploratory, it was not necessary to formulate sum scores among competing scales.

Consideration of factor loadings alongside item explained common variance (IECV) occurred at item level. According to Stucky and Edelen (2014), IECV reflects the extent to which an item represents a general factor, with values > 0.5 implying greater weighting for a general rather than a specific bifactor (Winebrake et al., 2024). To assess correlation strength between latent factors, the authors used the criteria of Gignac and Szodorai (2016), i.e., 0.10, 0.20, and 0.30 represented small, typical, and large associations.

## Results

### Model test

Assessment of the bifactor ESEM solution revealed satisfactory fit across indices, *χ*^2^(225) = 831.24, *p* < 0.001, CFI = 0.97, TLI = 0.96, RMSEA = 0.05 (0.04, 0.05), SRMR = 0.02. Bifactor specific criteria supported the existence of a general non-cognitive dimension underpinning the measures. Explicitly, the general factor possessed a *ω_h_* of 0.82, ECV of 0.64, and PUC of 0.68. Moreover, FD of 0.95 and H of 0.93 existed. Satisfactory coefficient *ω* existed for the bifactors (Mental Toughness *ω* = 0.88, Optimal Regulation *ω = 0*.74, Self-Efficacy *ω = 0*.92). However, relative *ω* inferred that each bifactor possessed a fairly low quantity of variance independent of the general dimension (Mental Toughness = 0.09, Optimal Regulation = 0.43, Self-Efficacy = 0.39).

Scrutiny of target-rotated standardized item loadings (Table 1) supported these observations. Mental Toughness exhibited greater loadings on the general vs. specific bifactor (mean loading of 0.57 vs. 0.18), as did Optimal Regulation (mean loading of 0.42 vs. 0.36) and Self-Efficacy (mean loading of 0.56 vs. 0.45). Moreover, 73% of items possessed IECV > 0.5, and IECV < 0.5 existed only for Mental Toughness negatively phrased items (2, 3, 6, and 7), Optimal Regulation items 3 and 4, and Self-Efficacy item 6.

**Table 1:** Bifactor ESEM factor loadings and Item Explained Common Variance (IECV).

Scale	Sub-scale	Item	General factor	Bifactor			IECV
				MTQ10	OR	GSES	
MTQ10		Even when under considerable pressure I usually remain calm.	0.71	0.01			1.00
		I tend to worry about things well before they actually happen.	0.32	0.71			0.17
		I usually find it hard to summon enthusiasm for the tasks I have to do.	0.31	0.42			0.35
		I generally cope well with any problems that occur.	0.78	−0.11			0.98
		I generally feel that I am a worthwhile person.	0.69	−0.12			0.97
		“I just do not know where to begin” is a feeling I usually have when presented with several things to do at once.	0.35	0.46			0.37
		When I make mistakes, I usually let it worry me for days after.	0.39	0.69			0.24
		I generally feel in control.	0.75	0.01			1.00
		I am generally able to react quickly when something unexpected happens.	0.70	−0.20			0.92
		I generally look on the bright side of life.	0.67	0.01			1.00
ER89-R	OR	I get over my anger at someone reasonably quickly.	0.37		0.23		0.72
		My daily life is full of things that keep me interested.	0.58		0.24		0.84
		I usually think carefully about something before acting.	0.40		0.54		0.34
		Most of the people I meet are likable.	0.26		0.29		0.42
		I quickly get over and recover from being startled.	0.46		0.43		0.53
		I am generous with my friends.	0.46		0.43		0.52
GSES		I can always manage to solve difficult problems if I try hard enough.	0.53			0.50	0.53
		If someone opposes me, I can find the means and ways to get what I want.	0.30			0.25	0.58
		It is easy for me to stick to my aims and accomplish my goals.	0.51			0.37	0.66
		I am confident that I could deal efficiently with unexpected events.	0.65			0.48	0.65
		Thanks to my resourcefulness, I know how to handle unforeseen situations.	0.57			0.51	0.55
		I can solve most problems if I invest the necessary effort.	0.49			0.61	0.40
		I can remain calm when facing difficulties because I can rely on my coping abilities.	0.70			0.36	0.79
		When I am confronted with a problem, I can usually find several solutions.	0.59			0.46	0.62
		If I am in trouble, I can usually think of a solution.	0.64			0.54	0.58
		I can usually handle whatever comes my way.	0.67			0.47	0.67

MTQ10 = Mental Toughness, ER89-R = Ego Resiliency, OR = Optimal Regulation, GSES = Self-Efficacy.

Collectively, these results supported the presence of a general non-cognitive dimension. Although, there is some support for the uniqueness of the specific bifactors. Indeed, for Mental Toughness, reverse-keyed items exhibited greater loadings on the bifactor. Furthermore, Optimal Regulation item 4 evidenced a weak loading on the general factor (0.26) and a loading of 0.29 on the bifactor. Controlling for these items resulted in a stronger ECV of 0.71 and weaker relative bifactor *ω* for Mental Toughness (0.01) and Optimal Regulation (0.41). Nonetheless, since they were core items within the standardized measures, the authors retained these.

Adding EI to the factorial model resulted in weaker data-model fit across indices vs. the NCAR solution, *χ*^2^(458) = 1786.54, *p* < 0.001, CFI = 0.95, TLI = 0.94, RMSEA = 0.05 (0.05, 0.06), SRMR = 0.03. Moreover, weaker bifactor specific indices existed (ECV = 0.57, *ω_h_* = 0.79) though H and FD remained similar. Items that contributed most strongly to the general factor included items indexing utilization and regulation of emotions (Table 2). Indeed, these contributed meaningfully (i.e., IECV > 0.5). However, remaining items associated with appraisal/knowledge of emotions exhibited IECV < 0.5. Subsequent analyses excluded EI as the construct did not contribute sufficient meaningful variance to the NCAR model. Moreover, research supports EI from the BEIS10 as a unidimensional construct (see Vredeveld, 2018). The BEIS-10 was designed to have five factors, but due to its brevity, it is commonly interpreted as measuring one overall, general emotional intelligence score. Hence, we opted against incorporating the significant items of this measure (alluding to specific features of EI) in the predictive model.

**Table 2:** Bifactor ESEM factor loadings and Item Explained Common Variance (IECV).

Scale	Sub-scale	Item	General factor	Bifactor				IECV
				MTQ10	OR	GSES	BEIS10	
MTQ10		Even when under considerable pressure I usually remain calm.	0.47	0.40				0.57
		I tend to worry about things well before they actually happen.	−0.06	0.66				0.01
		I usually find it hard to summon enthusiasm for the tasks I have to do.	0.14	0.46				0.08
		I generally cope well with any problems that occur.	0.55	0.45				0.60
		I generally feel that I am a worthwhile person.	0.61	0.41				0.69
		“I just do not know where to begin” is a feeling I usually have when presented with several things to do at once.	0.04	0.60				0.01
		When I make mistakes, I usually let it worry me for days after.	0.01	0.67				0.01
		I generally feel in control.	0.55	0.45				0.60
		I am generally able to react quickly when something unexpected happens.	0.62	0.25				0.86
		I generally look on the bright side of life.	0.58	0.39				0.70
ER89-R	OR	I get over my anger at someone reasonably quickly.	0.55		0.13			0.94
		My daily life is full of things that keep me interested.	0.44		0.32			0.65
		I usually think carefully about something before acting.	0.49		0.42			0.57
		Most of the people I meet are likable.	0.40		0.20			0.79
		I quickly get over and recover from being startled.	0.53		0.38			0.66
		I am generous with my friends.	0.39		0.55			0.33
GSES		I can always manage to solve difficult problems if I try hard enough.	0.56			0.48		0.58
		If someone opposes me, I can find the means and ways to get what I want.	0.30			0.33		0.45
		It is easy for me to stick to my aims and accomplish my goals.	0.53			0.34		0.71
		I am confident that I could deal efficiently with unexpected events.	0.61			0.52		0.57
		Thanks to my resourcefulness, I know how to handle unforeseen situations.	0.61			0.52		0.58
		I can solve most problems if I invest the necessary effort.	0.59			0.54		0.55
		I can remain calm when facing difficulties because I can rely on my coping abilities.	0.54			0.44		0.60
		When I am confronted with a problem, I can usually find several solutions.	0.61			0.44		0.66
		If I am in trouble, I can usually think of a solution.	0.65			0.51		0.62
		I can usually handle whatever comes my way.	0.63			0.45		0.66
BEIS10		I know why my emotions change	0.37				0.56	0.31
		I easily recognize my emotions as I experience them	0.38				0.64	0.26
		I can tell how people are feeling by listening to the tone of their voice	0.36				0.64	0.25
		By looking at their facial expressions, I recognize the emotions people are experiencing	0.36				0.68	0.22
		I seek out activities that make me happy	0.64				0.10	0.98
		I have control over my emotions	0.46				0.14	0.92
		I arrange events others enjoy	0.55				0.03	0.99
		I help other people feel better when they are down	0.69				0.04	0.99
		When I am in a positive mood, I am able to come up with new ideas	0.77				−0.04	0.99
		I use good moods to help myself keep trying in the face of obstacles	0.72				0.03	0.99

MTQ10 = Mental Toughness; ER89-R = Ego Resiliency; OR = Optimal Regulation; GSES = Self-Efficacy; BEIS10 = Brief Emotional Intelligence Scale.

### Descriptive statistics and correlations

Skewness and kurtosis statistics were within the acceptable range of −2 to +2 (Byrne, 2010) (Table 3). Inspection of latent factor correlations demonstrated significant small to moderate associations among the bifactors. However, within the bifactor model, Mental Toughness correlated negatively with Optimal Regulation and Self-Efficacy. This occurred because of bifactor orientation after the general factor accounted for the variance. Explicitly, the positively phrased items loaded strongly on the general factor, while the negatively keyed items took precedence on the bifactor. Indeed, latent factor associations in the three-factor ESEM solution showed large positive intercorrelations without the general factor’s presence (i.e., Mental Toughness with Optimal Regulation *r* = 0.33, Mental Toughness with Self-Efficacy *r* = 0.49, Optimal Regulation with Self-Efficacy *r* = 0.60). This aligns with Denovan et al. (2024a,b), who revealed an effect specific to pairs of negative items within the MTQ10. Although these items did not coalesce to form a method factor, isolated from the remaining positively phrased items they potentially skew direction toward the inverse of Mental Toughness (specifically ‘Mental Sensitivity’, Clough et al., 2002).

**Table 3:** Descriptive statistics and correlations among the bifactors.

Variable	*M*	*SD*	Skew.	Kurt.	1	2	3
1. Mental toughness	3.30	0.63	−0.20	0.67		−0.17**	−0.26**
2. Optimal regulation	2.78	0.54	0.03	−0.24	0.33**		0.22**
3. Self-efficacy	2.95	0.52	−0.44	0.94	0.49**	0.61**	

Scale means. Below the diagonal are associations from a correlated ESEM to examine results without a general factor, above the diagonal are correlations from the bifactor ESEM; **p < 0.001.

### Predictive validity

**Figure 1:**
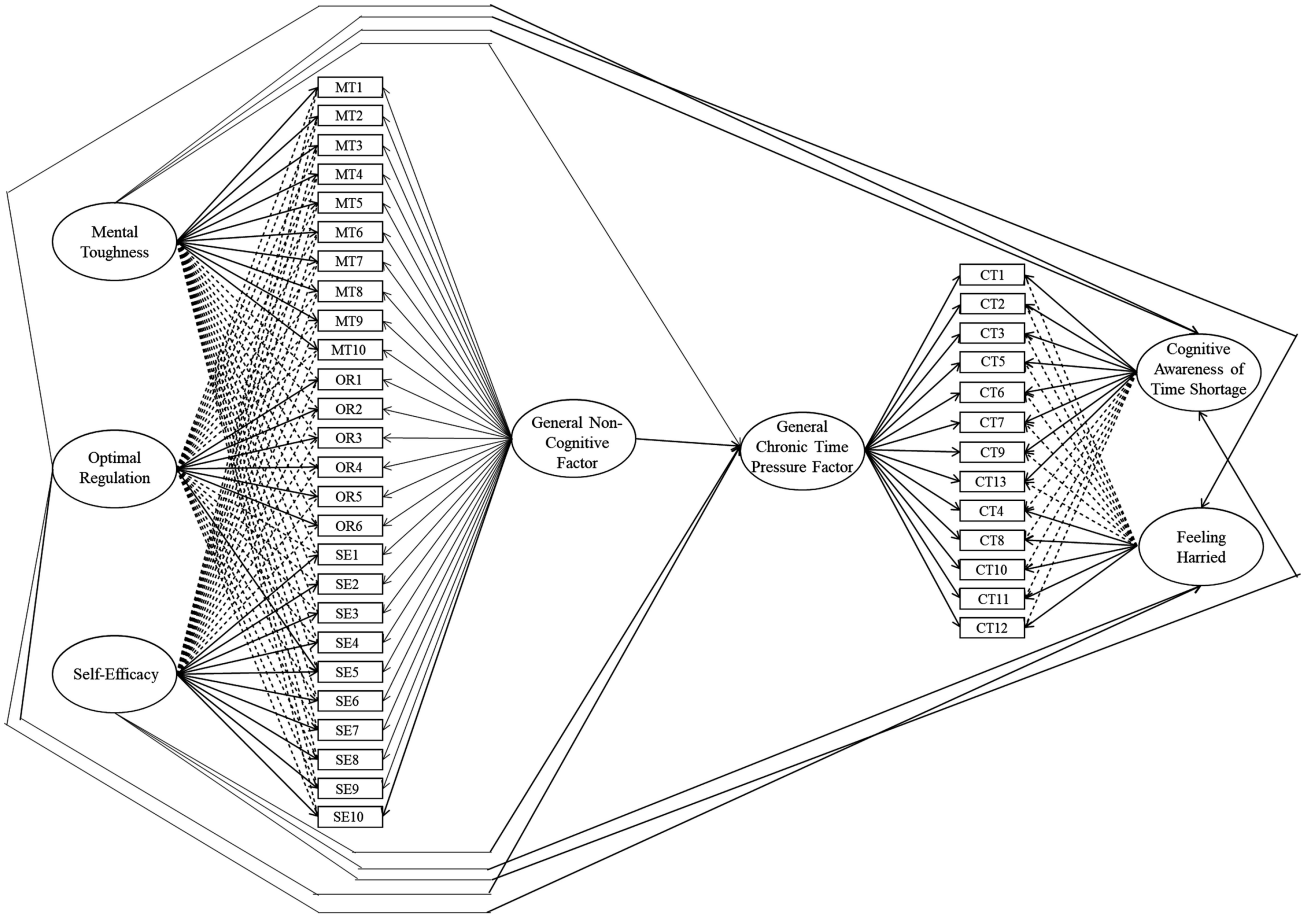
Schematic representation of the bifactor ESEM predictive model of chronic time pressure. Full unidirectional arrows represent factor loadings and predictive relations, dotted unidirectional arrows represent cross-loadings.

A model (see Figure 1 for a schematic representation) contrasting the effects of the general factor and specific bifactors from the bifactor ESEM, in relation to the criterion variable Chronic Time Pressure (CTP), revealed satisfactory fit, *χ*^2^(593) = 1706.56, *p* < 0.001, CFI = 0.96, RMSEA = 0.04 (0.04, 0.04), SRMR = 0.02. Mental Toughness (MT) was a significant negative predictor of both CA (*β* = −0.64, *p* < 0.001) and FH (*β* = −0.55, *p* < 0.001), as was Self-Efficacy (CA *β* = −0.19, *p* < 0.001; FH *β* = −0.18, *p* < 0.001).

Mental Toughness was also a significant negative predictor of the general CTP factor (*β* = −0.67, *p* < 0.001), whereas non-significant results existed for the remaining factors alongside the general non-cognitive factor. Optimal Regulation was a positive predictor of CA (*β* = 0.31, *p* < 0.001). The common variance among items captured by the general non-cognitive factor predicted FH negatively and significantly (*β* = −0.18, *p* < 0.001), and CA positively and significantly (*β* = 0.53, *p* < 0.001) and (*β* = −0.03, *p* = 0.251). The finding for CA was due to a mixed relationship with regards to Mental Toughness, Optimal Regulation, and Self-Efficacy.

These results indicated that the general non-cognitive dimension was a meaningful prognosticator of FH, with mixed results for both CA and the general CTP factor. The bifactor ESEM model predicted the outcomes (CA *R*^2^ = 0.81, FH *R*^2^ = 0.51, general CTP *R*^2^ = 0.46), indicating that the model possessed strong explanatory power.

## Discussion

This paper further assessed the psychometric properties of Non-Cognitive Adaptive Resourcefulness (NCAR), a recently developed non-cognitive model. This was necessary because following construct development validation comprised only a single study. Establishing model generalizability to an independent sample was therefore crucial to establishing NCAR as a psychological construct. Concomitantly, previous investigations limited nomological validity to perceived stress and anxiety control. While these represent important associated factors, to extend nomological validity, this study examined how NCAR related to Emotional Intelligence (EI), ensuring they can be meaningfully situated within a shared theoretical framework. Furthermore, to establish criterion validity, the study examined the model’s capacity to account for behavior in specific situations, namely Chronic Time Pressure (CTP).

Analysis replicated the bifactor latent structure reported previously. This comprised a general non-cognitive dimension underpinned by Mental Toughness, Optimal Regulation, and Self-Efficacy. Furthermore, low variance independent of the general dimension indicated that NCAR was a strong overarching construct. Accordingly, structure replication demonstrated the generalizability of the NCAR model and supported the supposition that the construct captures an underlying non-cognitive resource.

Regarding inclusion of Emotional Intelligence (EI) to the NCAR model, EI (versus the original solution) weakened data-model fit. Scrutiny of EI items revealed that statements associated with utilization (i.e., using emotions effectively) and regulation (i.e., managing emotions) contributed meaningfully to NCAR, whereas items related to appraisal (i.e., recognizing emotions) or knowledge (i.e., understanding emotions) did not add unique variance to the model. This observation was commensurate with the notion that NCAR embraces action-oriented and adaptive facets of emotional control and management, which are crucial for navigating challenges and achieving robust performance (see also Nguyen et al., 2019).

These EI facets represent key constituents of the broad non-cognitive factor, which encompasses motivation, perseverance, and effective coping strategies. Hence, how individuals use and regulate their emotions facilities beneficial capacities such as mental toughness (Qi, 2025), optimal self-regulation (Gómez-Leal et al., 2022), and self-efficacy (Chang and Tsai, 2022), thereby enhancing sense of control, belief in the ability to succeed, and resilience. This conceptualization aligns with the operationalization of NCAR as a positive, enabling resource that increases the capacity to maintain emotional equilibrium in demanding circumstances and thrive under pressure. The failure of cognitive/perceptual aspects (appraisal and knowledge) to contribute to the model suggests these components are either inherent within other aspects of NCAR (e.g., mental toughness, MT; Cowden, 2016; Kaiseler et al., 2009) or are not a central feature of the construct.

Overall, these results illustrated that since EI is a complex, multifaceted construct, its relationship with NCAR varies as a function of sub-factor. However, it is important to note that these findings are specific to the BEIS-10 and the current bifactor-ESEM specification. To confirm the generalizability of these structural relationships further research using more comprehensive EI measures is required. Hence, further research should use longer measures of EI, which provide factor scores. A notable example being the Schutte Self-Report Emotional Intelligence Test (Schutte et al., 1998). Although the original Schutte et al. (1998) paper proposed a unidimensional structure, subsequent studies have reported various multidimensional structures (e.g., Arunachalam and Palanichamy, 2017; Hallit et al., 2023; Petrides and Furnham, 2000; Saklofske et al., 2003).

The most common being a four-factor model that aligns with the theoretical framework of Salovey and Mayer (1990). This comprises perceiving emotions (i.e., identifying them in self and others), using emotions to facilitate thought (i.e., integrating them into cognitive processes), understanding emotions (i.e., comprehending their relationships and progression), and managing emotions effectively (i.e., regulating them in self and others for growth). Thus, subsequent investigations using dimensions of EI will provide theorists with a nuanced understanding of the interaction between NCAR and EI factors within a broad nomological network.

Regarding specific measurement decisions, we included all items despite some exhibiting low item explained common variance (IECV) values (see Table 2). We chose this approach to preserve the theoretical content validity and psychometric integrity of the established scales (BEIS-10 and MTQ10). Keeping the original scale structures allowed us to capture the full breadth of the constructs. The bifactor-ESEM approach then prevented specific item variance from biasing the general NCAR factor.

Using Chronic Time Pressure (CTP) to test the criterion validity of NCAR and its component constructs, we found that NCAR, MT, and Self-Efficacy (SE) predicted lower levels of Feeling Harried (FH). This indicated that individuals with higher NCAR, MT, and SE were less likely to experience the affective sensations of feeling rushed or overwhelmed by time demands. Findings for Cognitive Awareness of Time Shortage (CA) and overall CTP were complex. While MT and SE prognosticated lower CA, Optimal Regulation (OR) positively predicted CA.

To ensure the mixed predictive findings (where the general NCAR factor relates negatively to FH but positively to CA) represent a functional psychological response rather than a model artifact these outcomes require further consideration. We define these facets as representing the distinction between affective strain (FH) and cognitive appraisal (CA). From this perspective, the positive path to CA is adaptive; highly resourceful individuals maintain a realistic and heightened awareness of time constraints. This cognitive appraisal (CA) is a functional component of self-management and active coping, allowing individuals to acknowledge limited time as something to manage without acceding to emotional distress (FH). This mixed pattern suggests that NCAR facilitates a calm awareness, whereby individuals objectively recognize time constraints (high CA) without succumbing to the emotional distress (low FH) typically associated with temporal demands.

Furthermore, the positive CA path may be sensitive to the bifactor orientation and/or MTQ10 wording effects. By utilizing a bifactor ESEM approach, we separated the general adaptive variance of NCAR from specific factor noise, such as the “Mental Sensitivity” associated with negatively keyed items. This ensures the model captures a genuine relationship where NCAR facilitates realistic awareness (CA) as a prerequisite for the strategic deployment of resources.

Consistent with this, OR appears to function as an internal executive mechanism. This supposition is consistent with the delineation of OR as a process that allows for stable and adaptive functioning to life experiences. Thus, OR enables individuals to maintain balance when faced with stress, adjust to situational demands, realistically appraise challenging situations, and strategically deploy resources. These objective qualities foster realistic understanding of actual time limitations, which is a necessary feature of effective time management and self-management. Specifically, OR provides a strategic awareness of time that imparts a sense of control. Furthermore, MT negatively influenced the general CTP factor, while NCAR’s common variance positively influenced it. These findings suggest that NCAR emphasizes emotional and coping-related facets of time perception (i.e., FH) more than appraisal of time constraints (i.e., CA).

### Limitations

While this paper makes valuable contributions to scholarly understanding of non-cognitive skills, the authors acknowledge limitations. Firstly, the cross-sectional design prevents causal inferences about relationships between NCAR, EI, and CTP. Noting this, proceeding investigations should undertake longitudinal studies that enable theorists to assess the stability of the relationships outlined within this paper and evaluate the long-term influence of NCAR on chronic time pressure. This is important since this and preceding studies suggest that nurturing NCAR through training may enhance coping and stress resistance. Thus, research conducted over extended time periods could inform the development of interventions.

Secondly, this study comprised self-report measures. Although, the researchers used psychometrically attested instruments within an established methodological framework, self-report measures remain susceptible to response bias. This is true even when, as was employed in this study, researchers use procedural remedies (e.g., instruct participants to respond genuinely). In the context of non-cognitive skills and stress perception, this may manifest as social desirability, where respondents consciously or unconsciously project themselves as effectual copers.

Alternatively, respondents may under or overestimate their ability to deal with and resist stress. This is a common concern with self-report measures since participants are making subjective appraisals, which may not accurately reflect their capabilities. To counter these potential issues studies could include honesty checks and combine self-report with alternative (e.g., proxy judgments from colleagues) and objective measures including physiological indicators (e.g., heart rate variability, cortisol levels) and/or behavioral observations in simulated or real-world tasks that assess coping and self-management skills. This integrated approach will provide a comprehensive understanding of the interaction between NCAR and stress.

Thirdly, though this study used a large sample, it was UK-based. A range of external factors (i.e., social and policy environments) influence non-cognitive skills (Denovan et al., 2024a; Heckman and Kautz, 2012). Moreover, the sample was predominantly White, which may limit the generalizability of findings to more diverse populations. Hence, future studies should examine the extent to which the findings of this study extrapolate to other populations. This could include cross-cultural comparisons, which will deepen understanding of how non-cognitive skills develop and impact well-being across societal contexts and stressors. Theorists have commonly employed this model to assess the robustness of findings and constructs (Papageorgiou et al., 2023; Plouffe et al., 2023).

Fourth, conceptual rather than psychometric grounds informed our decision to retain MT and OR items with weaker item explained common variance (IECV) values. Specifically, these items were core components of the composite constructs. This approach benefited the study by maintaining consistency with prior work and enabling direct comparison. However, since it introduces minor specific factor variation it is suboptimal from a measurement perspective. Acknowledging this, future investigations should explore how best to refine NCAR items.

Finally, the finding that Emotional Intelligence did not meaningfully contribute to the NCAR model warrants further investigation. It is possible that the BEIS-10 measure, or its inherent conceptualization of EI did not align with the adaptive resourcefulness captured by the NCAR. Future research could explore alternative EI measures and further consider the facets of EI related to NCAR. Identifying intersections and distinctions between NCAR and allied non-cognitive constructs is an important, iterative aspect of conceptual advancement.

## Data Availability

The raw data supporting the conclusions of this article will be made available by the authors, without undue reservation.
