# Supplementary material for: Further assessment of the non-cognitive adaptive resourcefulness model comprising mental toughness, resilience, and self-efficacy: relationships with emotional intelligence and chronic time pressure
Source: Front Psychol. 2026 Feb 24;17:1718213. doi: 10.3389/fpsyg.2026.1718213 (PMC12971685; doi:10.3389/fpsyg.2026.1718213)
Supplement: Supplementary file 1 [file Table_1.DOCX]

Table S1. Descriptive statistics for Emotional Intelligence, Chronic Time Pressure and its Subscales

| Variable | *M* | *SD* | Skew. | Kurt. |
| --- | --- | --- | --- | --- |
| Emotional Intelligence | 3.37 | .50 | -.21 | .19 |
| Chronic Time Pressure | 3.24 | .70 | -.24 | .16 |
| Cognitive Awareness of Time Shortage | 2.07 | .28 | .26 | 1.48 |
| Feeling Harried | 3.20 | .84 | -.17 | -.30 |

*Note.* Scale means.
